# Enhancing Implant Prosthodontics: In Vitro Accuracy of Coded Healing Abutments on the Edentulous Lower Jaw

**DOI:** 10.1111/cid.70036

**Published:** 2025-05-09

**Authors:** Boldizsár Vánkos, Dénes Palaszkó, Kata Kelemen, Anna Németh, Judit Schmalzl, Dániel Márk Zentai, Elek Dinya, Péter Hermann, Barbara Kispélyi

**Affiliations:** ^1^ Department of Prosthodontics Semmelweis University Budapest Hungary; ^2^ Institute of Digital Health Sciences, Semmelweis University Budapest Hungary

**Keywords:** dental casting technique, dental implantation, dental impression technique, models, prosthodontics

## Abstract

**Objective:**

This study aimed to investigate the accuracy of conventional and digital impression‐making and cast‐fabrication using coded healing abutments on an edentulous mandibular model under in vitro conditions.

**Materials and Methods:**

Our study investigated the accuracy of the On1 Concept (Nobel Biocare; Kloten, Switzerland) coded healing abutment system using conventional and digital workflows. The Conical Connection (CC) system (Nobel Biocare; Kloten, Switzerland) was used as the control group in both workflows. 10–10 open‐tray impressions and intraoral scans were made from the reference model with each system. Models built from intraoral scans were additively fabricated, and open‐tray impressions were poured with type‐4 dental stone. The prepared models were digitized using a desktop scanner with an accuracy of 4 μm (E4, 3Shape; Copenhagen, Denmark) and superimposed on the reference scan. Four linear distances and root mean square (RMS) deviations were measured using metrology software.

**Results:**

Five dimensions were measured using signed and absolute deviations, resulting in nine outcomes. RMS and diagonal deviations provided the most insight into overall model deviations. Mean RMS deviations were: 58.30 (14.95) μm for CC_conv, 47.66 (13.04) μm for On1_conv, 204.97 (37.40) μm for CC_dig, and 136.64 (13.49) μm for On1_dig. Significant differences were found between On1_conv vs. CC_dig, On1_conv vs. On1_dig, and CC_conv vs. CC_dig. Mean linear deviations between the molar positions were: 24.49 (58.20) μm for CC_conv, 87.46 (106.70) μm for On1_conv, −104.76 (125.83) μm for CC_dig, and 140.64 (190.56) μm for On1_dig. Significant differences were observed between On1_conv vs. CC_dig and CC_dig vs. On1_dig.

**Conclusions:**

Based on the RMS deviations, the conventional method is significantly more accurate at both implant and platform levels in the case of an in vitro edentulous lower jaw model. The RMS deviations of the implant analogs are smaller on the platform level with both conventional and digital methods.

## 1 Introduction

Precise impression‐making of an edentulous lower jaw is one of the biggest challenges in implant prosthodontics. Traditionally, the open tray impression method is considered to be the gold standard of implant impressions [1]. Although being a widely used, well‐proven method, open‐tray impression‐making has its limitations. The process consists of several clinical and laboratory steps (including the splinting of the impression‐copings, fabrication‐ and adjustments of the special tray, mixing the impression material and pouring the impression with gypsum) thus carrying many possibilities for mistakes.

Digital impression‐making offers several advantages over conventional methods, particularly in terms of treatment time, patient comfort, and workflow efficiency [2]. Intraoral scanning minimizes errors associated with material distortions and manual handling, providing a more reliable and predictable workflow [3, 4]. By eliminating the need for impression trays and the setting time of impression materials, this approach significantly improves patient comfort [5]. The streamlined digital workflow reduces chair time and facilitates faster and more convenient communication with dental laboratories, accelerating the overall treatment process [6]. Moreover, the ability to provide three‐dimensional visualizations enhances treatment planning and helps patient communication [7]. Despite these advantages, challenges such as the accuracy of full‐arch digital scans and the need for adequate operator training persist [8].

Coded healing abutments (CHA) combine the functions of healing abutments, impression copings or scanbodies, and sometimes even temporary prosthetic abutments. Several systems with different designs are available on the market. There are one‐piece scannable healing abutments, multi‐piece modular systems with impression copings, scanbodies that attach to the healing abutment, and systems with a titanium base or platform that can carry all the prosthetic abutments, thus elevating the implant interface from the bone level.

**FIGURE 1:**
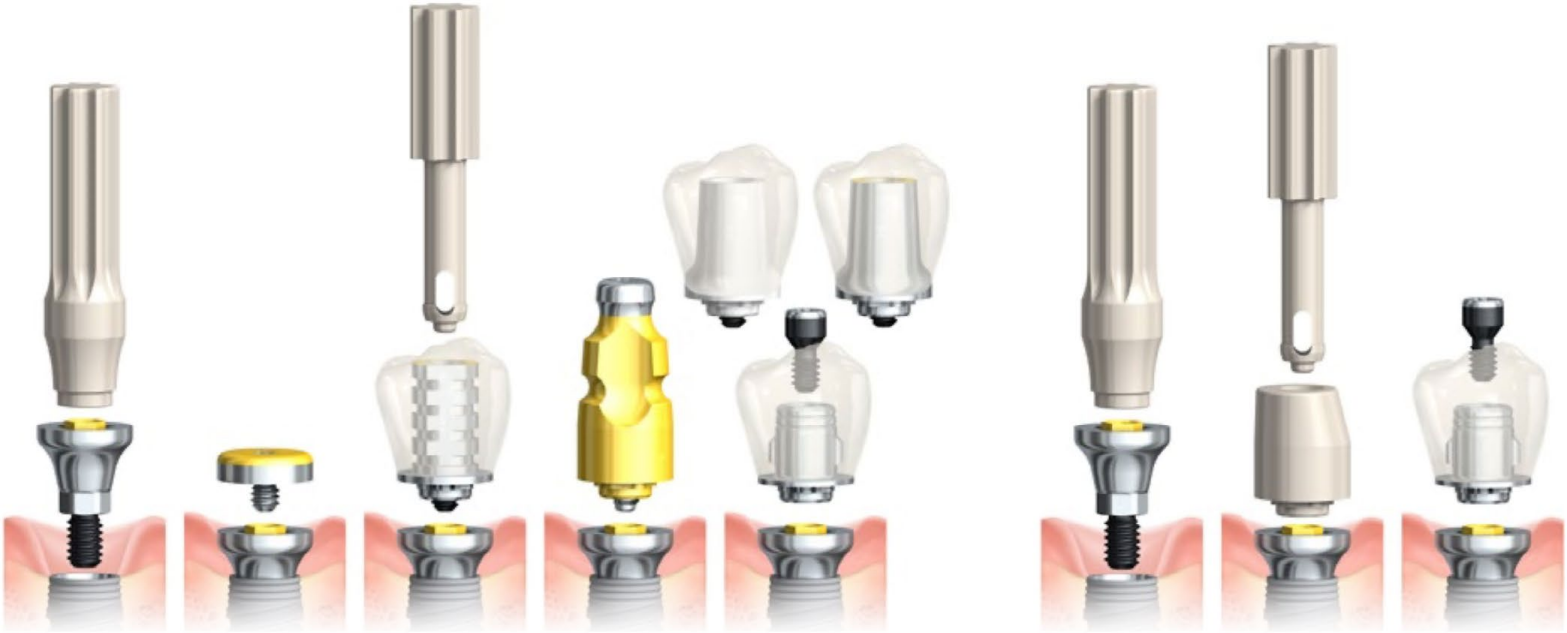
Prosthetic workflow of the On1 concept (Nobel Biocare; Kloten, Switzerland).

One‐piece CHAs are usually inserted at implant placement and are only removed once the final prostheses are delivered. Such systems are, for example, the Profile Designer iPhysio (Lyra ETK; Sallanches, France) or the Encode Emergence Healing Abutment (ZimVie; Westmisnter, USA). Multi‐piece systems usually have a platform or base (in most cases made out of titanium) that allows us to detach and attach different parts, such as healing caps, impression copings, or scan bodies, without damaging the connective tissue and junctional epithelium. Such a system is, for example, On1 Concept (Nobel Biocare; Kloten, Switzerland). In Figure 1, the prosthetic workflows of the On1 system can be seen in both conventional and digital impression making. All the prosthetic parts, such as healing caps, impression copings, scan bodies, and prosthetic abutments, attach to the On1 base platform without having to remove them.

Coded healing abutments have many advantages compared to conventional replaceable systems. They make the prosthetic workflows quicker and more straightforward, reducing the time spent in the dental chair [9]. However, the main advantage of these systems is that, in contrast with the conventional method, the peri‐implant soft tissue environment is not disturbed during and after the healing and maturation processes. Studies have found more favorable bone‐loss outcomes associated with CHA than with conventional abutment systems; however, the clinical relevance of these data is questionable [10, 11]. Mouhyi et al. found CHAs to be clinically reliable while providing high prosthetic precision and esthetics and promoting hard and soft tissue stability [12].

Thanks to the advantages mentioned above, coded healing abutments are becoming increasingly popular and widely used. However, long‐term data regarding the accuracy of prosthetic workflows using these systems are not yet available.

In the case of implant‐retained prostheses, accuracy plays a crucial role. Unlike natural teeth, Implants are usually captured indirectly by conventional or digital impressions. The physical or virtual implant analogs in the physical or virtual cast represent the position of the implants in the jaw.

If analogs (either digital or conventional) are not perfectly aligned with the original implant positions, prosthesis misfit threatens the long‐term success of the implants and prostheses. Misfit and consequential tension and stress can increase the risk of mechanical and biological complications such as screw loosening, screw fracture, ceramic chipping, and marginal bone loss [5].

The objective of this study was to investigate the accuracy of different impression‐making and cast fabrication methods, including conventional and CHA systems, using an in vitro edentulous lower jaw model. We aimed to help clinicians navigate through the different impression‐making techniques based on evidence, allowing them to choose the best for their patients.

Our hypothesis is that the conventional platform‐level open‐tray impression technique demonstrates superior accuracy compared to digital impression methods in the context of edentulous full‐arch implant rehabilitation.

## 2 Materials and Methods

### 2.1 Test Groups and Workflow

**FIGURE 2:**
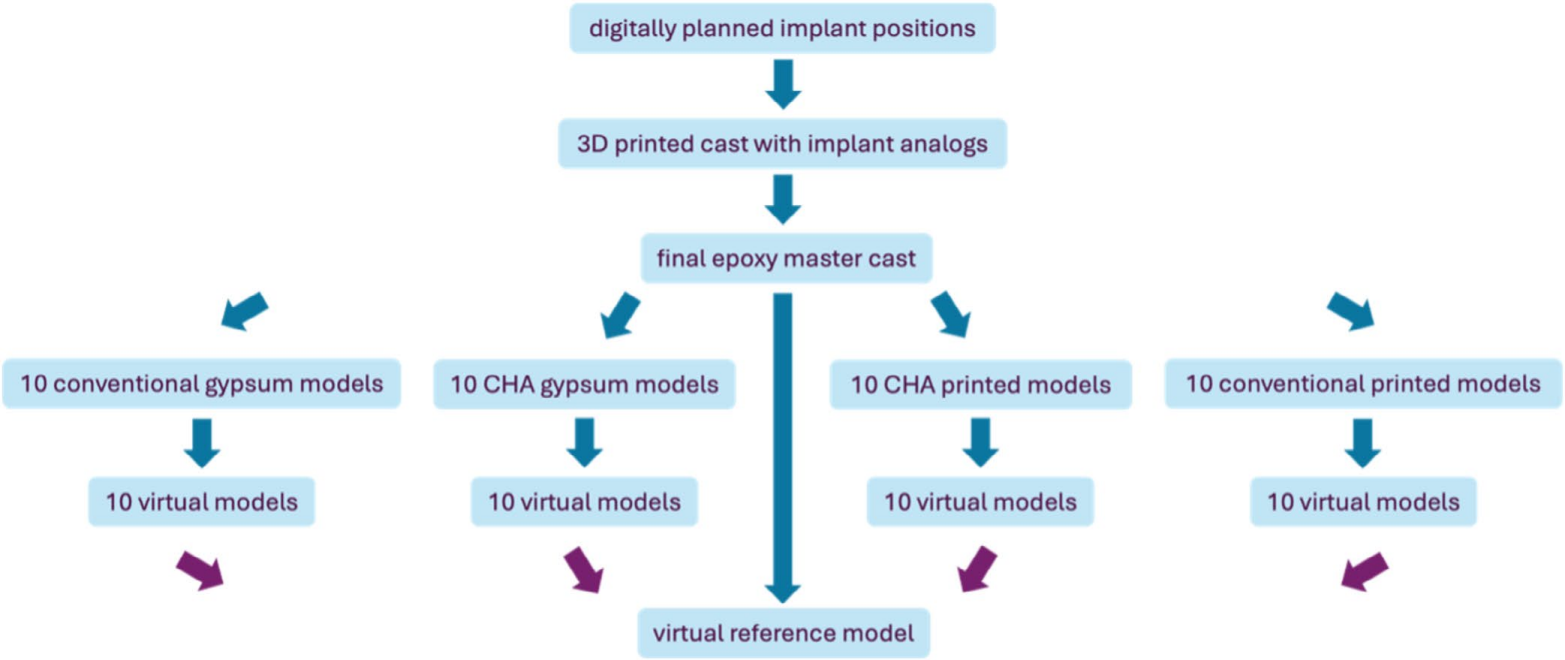
Workflow of the study.

Four test groups were established to compare the accuracy of the different methods. The groups respectively are the following: conventional open‐tray impression method using the Conical Connection system (Nobel Biocare; Kloten, Switzerland) ***(CC_conv)***, digital impression‐making method with intraoral scanner using the Conical Connection system (Nobel Biocare; Kloten, Switzerland) *(CC_dig)*, conventional open‐tray impression method using the On1 system (Nobel Biocare; Kloten, Switzerland) **(On1_conv)**, digital impression‐making method with intraoral scanner using the On1 system (Nobel Biocare; Kloten, Switzerland) ***(On1_dig)***. The workflow of the study can be seen in Figure 2.

### 2.2 Master Model

A master model was created from an edentulous mandibular typodont. First, four implant positions were designed in SMOP Swissmedia software (Swissmedia; Baar, Switzerland) to replicate an all‐on‐4 status [13]. Two NobelReplace CC tapered regular platform implants (Nobel Replace; Klonte, Switzerland) were designed vertically, parallel to each other in the interforaminal section of the jaw, whereas the two distal implants were planned at an angle of 40°. The virtually planned implants were then digitally replaced with digital implant replicas using exocad software (exocad GmbH; Darmstadt, Germany) to create a virtual model with implant‐replica sockets corresponding to the planned implant positions.

Around the coronal middle point of the analogs, a sphere with a 5 mm radius was cut out to allow the different abutment designs to fit on the cast without having to use artificial gingiva. The planned master model was additively fabricated using Pro 4K (Asiga; Sydney, Australia), and the conical connection, implant‐level analogs were inserted subsequently. An open‐tray impression of the printed cast was made and poured with epoxy resin. This way, we replicated the original design in a sturdy and stable model, which was then appointed as the final master cast and baseline for later measurements. For later measurements, the physical master model was digitized with both implant‐level Elos Accurate Scan Bodies (Elos Medtech; Goteborg, Sweden) and platform‐level On1 IOS Healing Caps (Nobel Biocare; Kloten, Switzerland) attached using a laboratory‐grade desktop scanner.

### 2.3 Conventional Methods

#### 2.3.1 Impression‐Making

**FIGURE 3:**
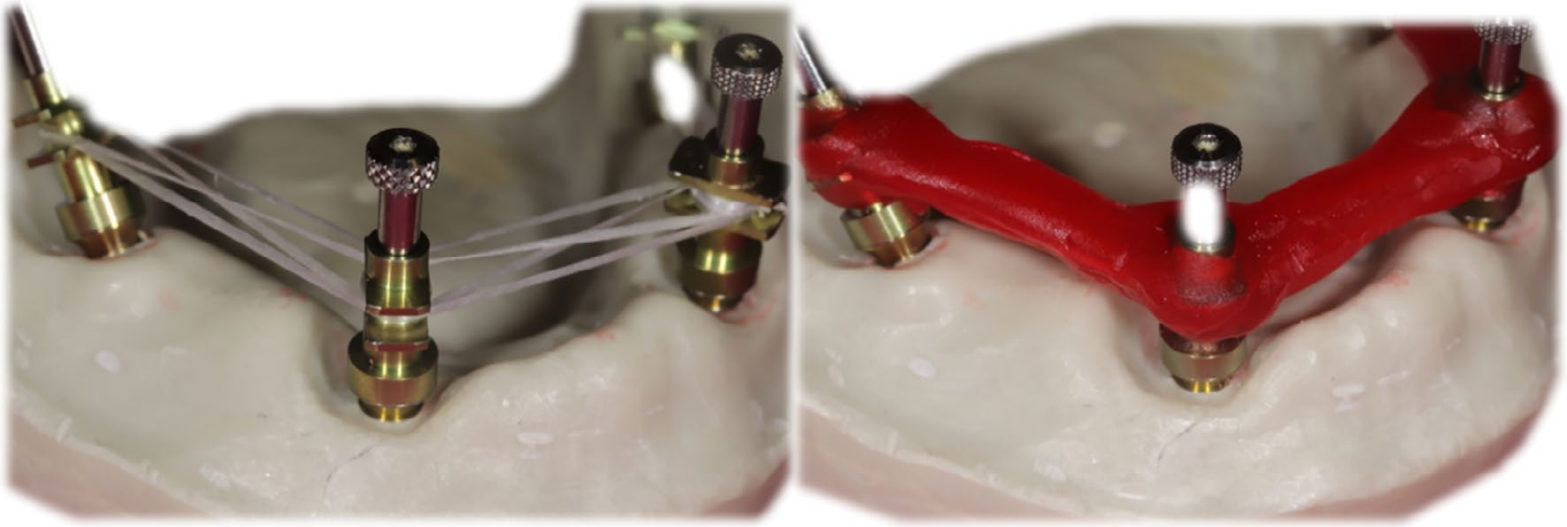
Splinting of the impression copings.

**FIGURE 4:**
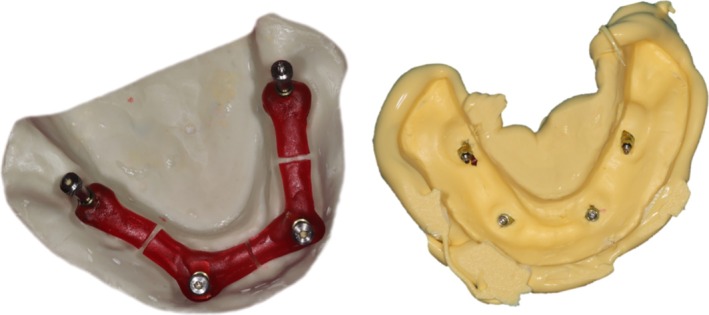
Separation, reattachment, impression‐making.

For the conventional impression groups, implant level and platform level open tray impression techniques were used. In the CC_conv group, implant level open tray impression copings (Nobel Biocare; Kloten, Switzerland) were attached, and in the On1_conv group, On1 platforms (Nobel Biocare; Kloten, Switzerland) platform level open tray impression copings (Nobel Biocare; Kloten, Switzerland) were used. The copings were attached to the analogs, and the screws were tightened with standard torque. The copings were splinted using dental floss and self‐curing acrylate (GC International AG; Luzern, Switzerland) (Figure 3) The splints were then separated (Figure 4) with thin disks and rejoined with a small amount of resin to avoid the deformation caused by shrinkage.

The master model was scanned together with an impression splint, and a special tray was designed on this scan file using Blender (Blender Foundation; Amsterdam, Netherlands) software. The special tray was designed to ensure a uniform layer thickness for the impression material around the cast and to include the outside edge of the cast all around. The tray was additively fabricated using Pro 4 K (Asiga; Sydney, Australia). The monophase technique was used for the impressions. After applying Universal tray adhesive (Zhermack; Badia Polesina, Italy), Elite HD+ maxi monophase (Zhermack; Badia Polesina, Italy), a machine‐mixed additional silicone impression material was used (Figure 4).

#### 2.3.2 Cast‐Fabrication

After the impression‐making, the appropriate implant‐ or platform‐level implant analogs were connected to the impression copings and tightened by hand. The impressions were poured the same day with type 4 dental stone. The gypsum was mixed and handled according to the manufacturer's instructions. Excess dental stone was trimmed so that the outside edge of the cast remained visible.

### 2.4 Digital Methods

#### 2.4.1 Impression‐Making

**FIGURE 5:**
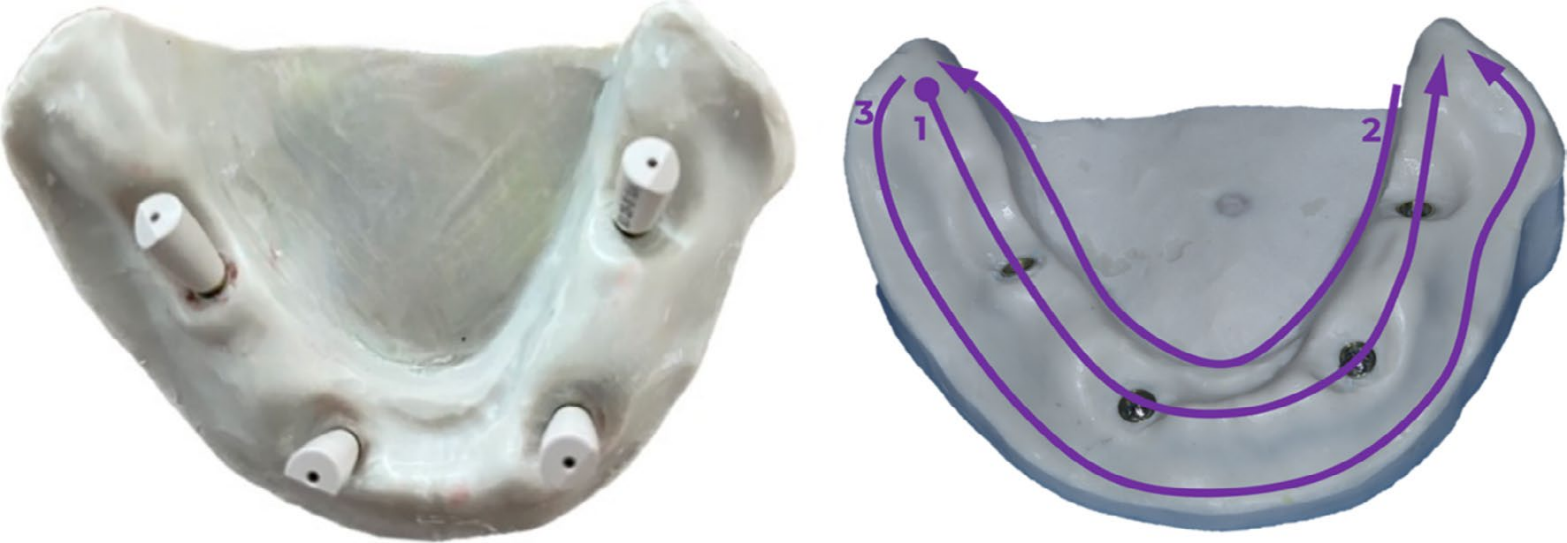
Scanbodies on the model and scanning strategy.

For the digital groups, implant‐ and platform‐level scanning was performed. In the CC_dig group, implant‐level Elos Accurate Scan Bodies (Elos Medtech; Goteborg, Sweden) (Figure 5) were used; in the On1_dig group, platform‐level On1 IOS Healing Caps (Nobel Biocare; Kloten, Switzerland) were attached and the screws were tightened with standard torque. For the scanning, a Trios 5 (3Shape; Copenhagen, Denmark) intraoral scanner was used. For each optical impression, the scanning started at the cast's right side. First, the occlusal surface of the alveolar ridge was scanned in a continuous line, including the precise scanning of the scanbodies. This was followed by the lingual and vestibular surfaces of the jaw and, finally, any missing parts (Figure 5) Every scan was conducted by the same operator, and the same scanning strategy was followed in every case. During scanning, ambient lighting was minimized.

#### 2.4.2 Cast Fabrication and Post‐Processing

For the fabrication of the casts in the digital groups, the scan files were imported into the 3Shape Dental System (3Shape; Copenhagen, Denmark), and models were designed with the appropriate digital implant replicas. The models were additively manufactured with Pro4k (Asiga; Sydney, Australia) using printodent GR‐13 model material (Pro3Dure; Iserlohn, Germany). The printer settings were standardized and adjusted based on the manufacturer's instructions. The models were arranged on the printing platform in batches of five and tilted at 22.5° based on the results of Lojo et al. [14].

The models were post‐processed according to the manufacturer's instructions and first washed in an ultrasonic isopropyl immersion cleaning unit—Ultrasonic Cleaner (Soundlin; Suzhou, China) for six minutes, followed by a water immersion bath—CLD1 (Pro3Dure; Iserlohn, Germany) for seven minutes. After washing, the models were air‐dried at room temperature with a compressed air duster and were polymerized for 4 min at 22°C using CLD2 (Pro3Dure; Iserlohn, Germany).

#### 2.4.3 Transportation, Handling, and Storage of the Finished Printed Models

The models were stored spaciously, flat on their base, protected from physical impacts and light. The ambient temperature of the storage unit was between 20°C and 24°C, and the humidity was between 54% and 61%. All printed models were digitized within one week to avoid inaccuracies caused by insufficient dimensional stability.

### 2.5 Digitization, Measurements

The master model was digitized using a laboratory‐grade desktop scanner E4 (3Shape; Copenhagen, Denmark) with an accuracy of 4 μm.

All models were digitized using the same laboratory‐grade desktop scanner with a precision scanning method around the scanbodies. For each model, the corresponding scanbodies were attached.

The master and test models were imported into Geomagic Control X (Oquton; North Carolina, United States) metrology software. The excess parts of the models were trimmed by hand in each case.

Best fit alignment was used to superimpose the test models on the corresponding master models. For the alignment, the surface of the models was selected except for the surface of the scanbodies. This ensures that the real differences are not masked by the alignment method.

Five measurements were conducted for each model: root mean square (RMS) deviations of the scanbodies and four linear distances.

**FIGURE 6:**
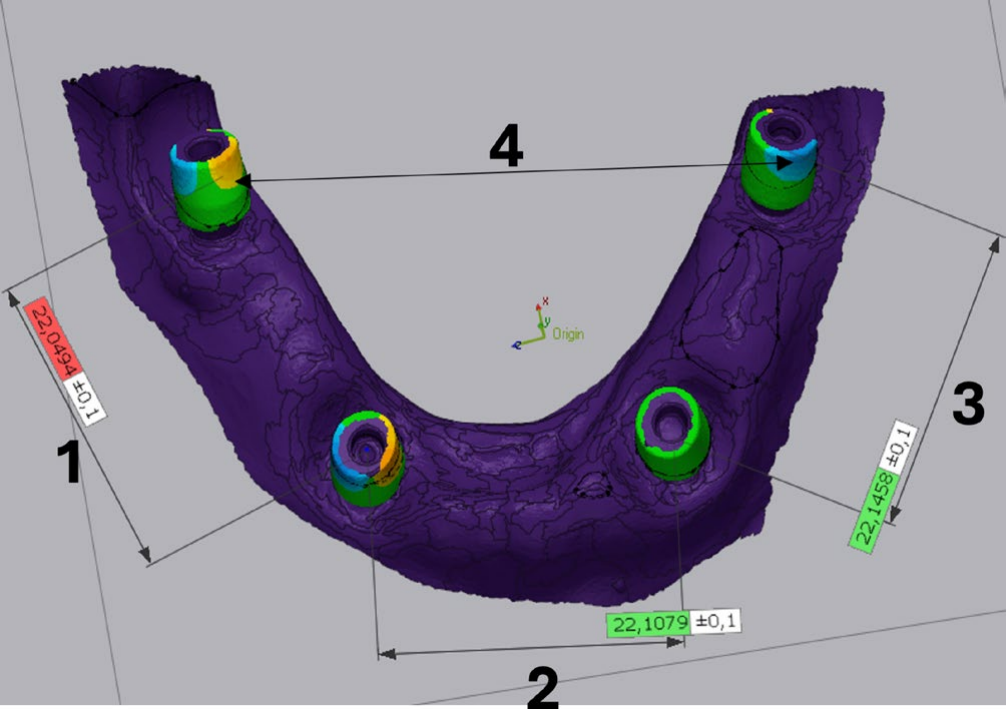
Measured distances on the virtual model.

For the linear distances, a cross‐section was taken across the scanbodies, parallel with the occlusal surface of the alveolar ridge. The plane's distance from the model surface was standard in all cases. ***Lin1_right***—the distance between the right molar and right canine positions, ***Lin2_front***—the distance between the two canine positions, ***Lin3_left***—the distance between the left canine and left molar positions, ***Lin4_diagonal***—the diagonal distance between the two molar positions. For every linear measurement, both the original signed deviations and the absolute deviations were measured. The absolute deviations were named **Alin1_right, Alin2_front, Alin3_left, and Alin4_diagonal**, respectively. For the linear distances, one point on the outer edge of each scanbody cross‐section was selected, creating the shortest possible distance between the cross‐section. The measurements were automated and standardized by creating a measurement template using only the reference file and one test file. After that, the measurement method was not modified, but files were added using the “batch process” function of the software (Figure 6).

### 2.6 Statistical Analysis

All statistical analyses were performed using SPSS Version 29 (IBM Corp., 2023). The continuous outcomes (superimposition tests) and descriptive statistics (sample size, mean, median, standard deviation, min., max.) were calculated for each group. The assumption of normality was assessed using the Shapiro–Wilk test, and the results indicated that the assumption was violated. Levene's test was used to assess the homogeneity of variance. Therefore, the nonparametric Kruskal–Wallis (1‐way ANOVA) test was used to determine the global significance of differences among the four groups, with a significance level of α = 0.05. Because of the large number of correlations, Bonferroni correction was used for post hoc comparisons. A median test was also conducted. All analyses were conducted based on the originally signed deviations and absolute deviations as well to avoid seemingly minor mean deviations caused by large positive and negative values canceling each other out.

## 3 Results

Data from the virtual measurements were used for descriptive statistics and analyses. Table 1 represents each group's mean, SD, min, max, N, and grouped median.

**TABLE 1:** Descriptive statistics for the examined groups.

Groups	Parameters	RMS	Lin1_right	Lin2_front	Lin3_left	Lin4_diagonal	Alin1_right	Alin2_front	Alin3_left	Alin4_diagonal
CC_conv	Mean	58.300	117.060	−11.280	24.030	−24.490	117.0600	24.9000	37.2100	51.2900
	*N*	10	10	10	10	10	10	10	10	10
	Std. Deviation	14.9527	59.6904	34.3835	40.3479	58.2020	59.69039	25.19321	27.03929	33.62897
	Minimum	32.7	35.7	−86.8	−36.5	−101.3	35.70	0.40	7.70	5.80
	Maximum	76.0	223.4	31.1	74.8	89.2	223.40	86.80	74.80	101.30
	Grouped Median	61.100	113.250	0.850	18.850	−36.700	113.2500	23.9000	31.9000	61.9000
ON1_conv	Mean	47.660	−7.190	−103.860	53.430	87.460	46.7900	103.8600	60.8700	126.2600
	*N*	10	10	10	10	10	10	10	10	10
	Std. Deviation	13.0407	55.1066	41.8033	37.0998	106.7044	25.72182	41.80333	20.77290	46.60606
	Minimum	29.6	−96.5	−187.3	−37.2	−108.5	15.80	33.70	37.20	69.90
	Maximum	64.4	50.1	−33.7	87.6	238.5	96.50	187.30	87.60	238.50
	Grouped Median	48.167	−6.050	−102.633	55.400	116.600	39.7667	102.6333	55.4000	116.6000
CC_dig	Mean	204.970	−136.530	37.930	147.140	−104.760	158.2500	40.0700	147.1400	138.1000
	*N*	10	10	10	10	10	10	10	10	10
	Std. Deviation	37.4045	123.1152	44.3036	82.7008	125.8269	89.68456	42.15849	82.70083	82.67894
	Minimum	159.7	−326.6	−4.7	32.4	−263.5	13.80	1.90	32.40	23.80
	Maximum	281.9	94.8	119.1	355.9	108.4	326.60	119.10	355.90	263.50
	Grouped Median	208.900	−188.350	30.350	136.450	−148.400	188.3500	30.3500	136.4500	148.4000
On1_dig	Mean	136.640	97.590	73.680	141.730	140.640	97.5900	74.1400	141.7300	188.8600
	*N*	10	10	10	10	10	10	10	10	10
	Std. Deviation	13.4898	47.2342	50.0975	57.2170	190.5617	47.23423	49.33766	57.21696	136.60116
	Minimum	117.3	22.1	−2.3	21.8	−135.5	22.10	2.30	21.80	1.40
	Maximum	157.9	163.3	153.4	219.6	386.8	163.30	153.40	219.60	386.80
	Grouped Median	136.750	104.100	73.000	152.900	171.350	68.46	35.21	69.96	109.64

### 3.1 Accuracy Outcomes—Root Mean Square Deviations

**FIGURE 7:**
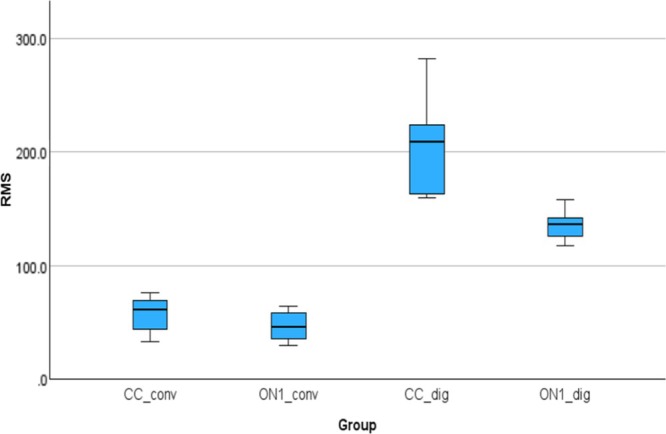
Independent sample Kruskal‐Wallis test of RMS deviations.

Figure 7 shows the root mean square deviations of the implant analogs in all groups compared to the reference model. The mean (SD) was 58.30 (14.95) μm for the CC_conv, 47.66 (13.04) μm for the On1_conv, 204.97 (37.40) μm for the CC_dig, 136.64 (13.49) μm for the On1_dig.

Using the nonparametric Kruskal‐Wallis test, a statistically significant difference was observed between On1_conv and CC_dig groups with a *p*‐value of 0.000, On1_Conv and On1_dig with a *p*‐value of 0.006, and between CC_Conv and CC_dig with a *p*‐value of 0.000.

### 3.2 Accuracy Outcomes—Lin1_right, Alin1_right

**FIGURE 8:**
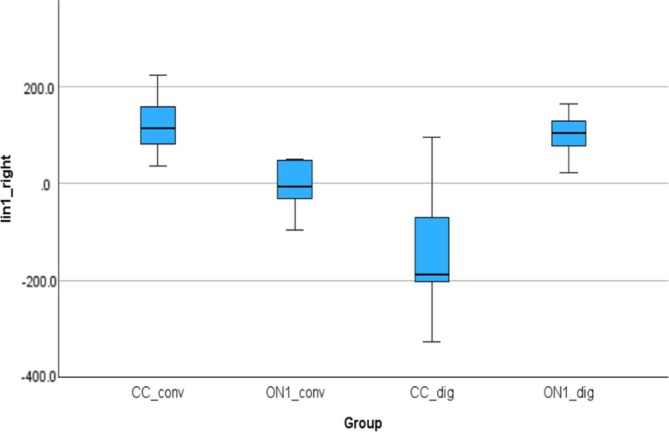
Independent sample Kruskal‐Wallis test of the linear deviations in Lin1_right distance.

Figure 8 shows the linear deviations between the analogs in the right molar and right canine positions. The mean (SD) was 117.06 (59.69) μm for the CC_conv, −7.19 (55.01) μm for the On1_conv, −146.53 (123.11) μm for the CC_dig, 97.59 (47.23) μm for the On1_dig. Using the nonparametric Kruskal‐Wallis test, a statistically significant difference was observed between CC_Conv and CC_dig groups with a *p*‐value of 0.001, between CC_conv and CC_dig with a *p*‐value of 0.000 and between CC_Conv and On1_dig with a *p*‐value of 0.0019.

**FIGURE 9:**
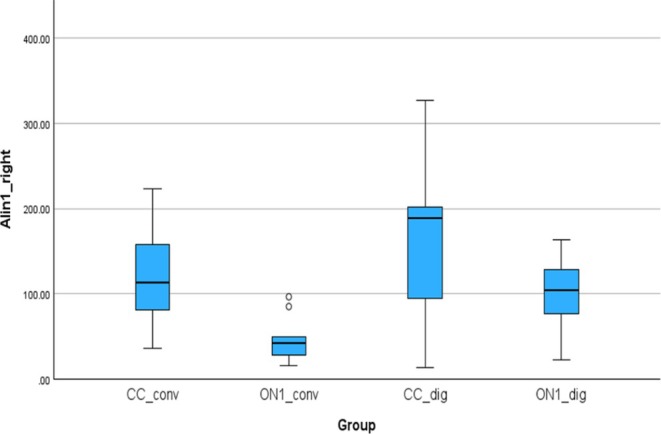
Independent sample Kruskal‐Wallis test of the absolute linear deviations in Lin1_right distance (Alin1_right).

Figure 9 shows the absolute linear deviations measured between the analogs in the right molar and right canine positions. The absolute mean (SD) was 117.06 (59.69) μm for the CC_conv, 46.79 (25.72) μm for the On1_conv, 158.25 (89.68) μm for the CC_dig, 97.59 (47.23) μm for the On1_dig. Using the nonparametric Absolute‐Kruskal‐Wallis test, a statistically significant difference was observed between On1_conv and CC_dig groups with a *p*‐value of 0.006.

### 3.3 Accuracy Outcomes—Lin3_left, Alin3_left

**FIGURE 10:**
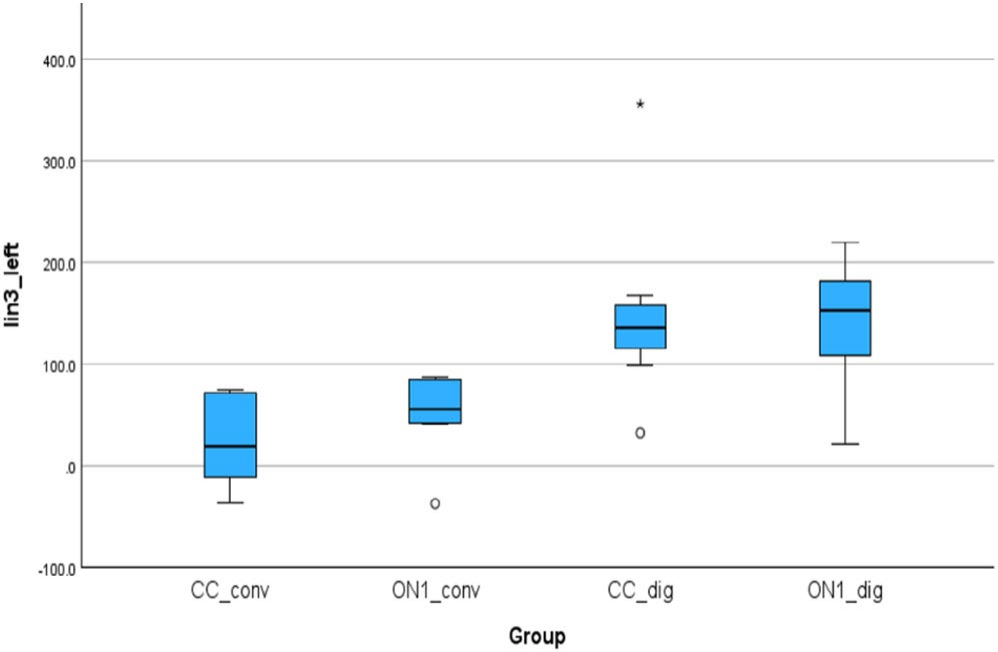
Independent sample Kruskal‐Wallis test of the linear deviations in Lin3_left distance.

Figure 10 shows the linear deviations measured between the analogs in the left molar and left canine positions. The mean (SD) was 24.03 (40.34) μm for the CC_conv, 53.43 (37.09) μm for the On1_conv, 147.14 (82.70) μm for the CC_dig, 141.73 (57.21) μm for the On1_dig. Using the nonparametric Kruskal‐Wallis test, a statistically significant difference was observed between the CC_dig and CC_Conv groups with a *p*‐value of 0.001, the CC_Conv and On1_dig with a *p*‐value of 0.000, and between the On1_Conv and On1_dig with a *p*‐value of 0.022.

**FIGURE 11:**
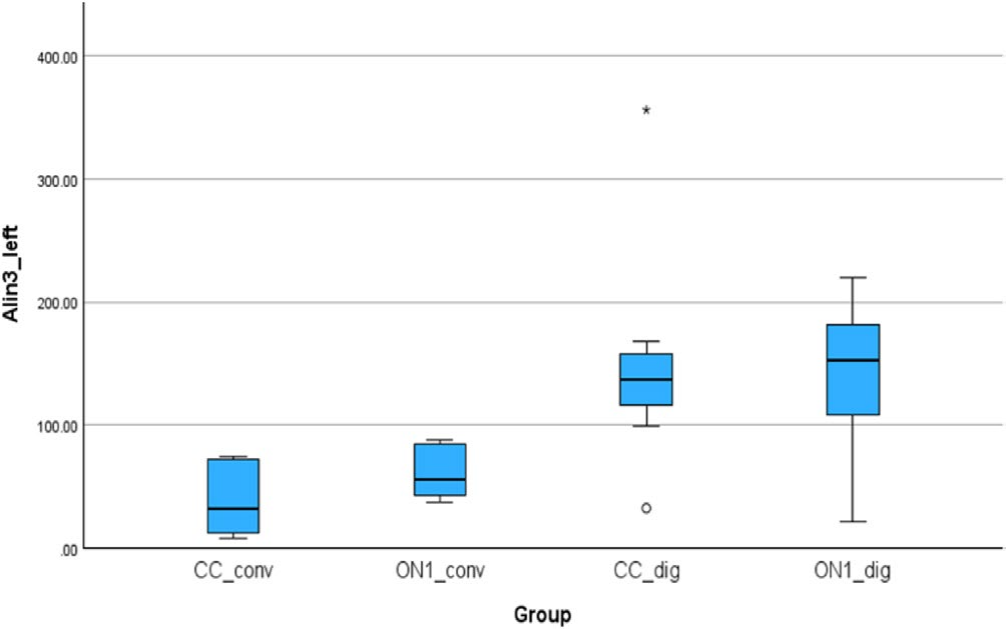
Independent sample Kruskal‐Wallis test of the absolute linear deviations in Lin3_left distance (Alin3_left).

Figure 11 shows the absolute linear deviations measured between the analogs in the left molar and left canine positions. The absolute mean (SD) was 37.21 (27.04) μm for the CC_conv, 60.87 (20.77) μm for the On1_conv, 147.14 (82.70) μm for the CC_dig, 141.73 (57.22) μm for the On1_dig. Using the nonparametric Absolut‐Kruskal‐Wallis test, a statistically significant difference was observed between the CC_conv and CC_dig groups with a *p*‐value of 0.001. between the CC_conv and On1_dig groups with a *p*‐value of 0.000 and between the On1_conv and On1_dig groups with a *p*‐value of 0.04.

### 3.4 Accuracy Outcomes—Lin2_front, Alin2_front

**FIGURE 12:**
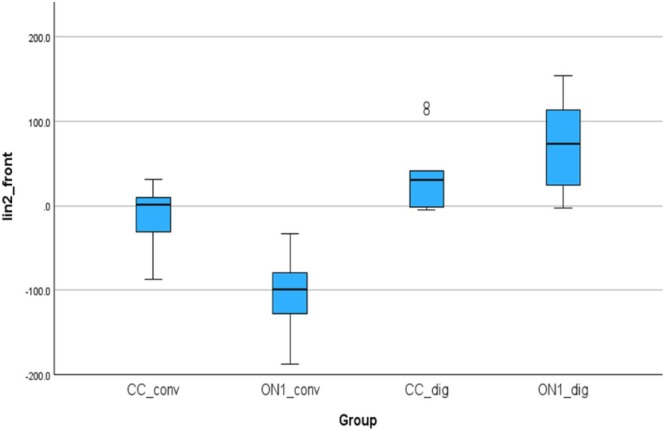
Independent sample Kruskal‐Wallis test of the linear deviations in Lin2_front distance.

Figure 12 shows the linear deviations measured between the analogs in the right and left canine positions. The mean (SD) was −11.28 (34.38) μm for the CC_conv, −103.86 (41.80) μm for the On1_conv, 37.93 (44.30) μm for the CC_dig, 73.68 (50.10) μm for the On1_dig. Using the nonparametric Kruskal‐Wallis test, a statistically significant difference was observed between the On1_conv and CC_dig groups, with a *p*‐value of 0.000 and between On1_Conv and On1_dig, with a *p*‐value of 0.000.

**FIGURE 13:**
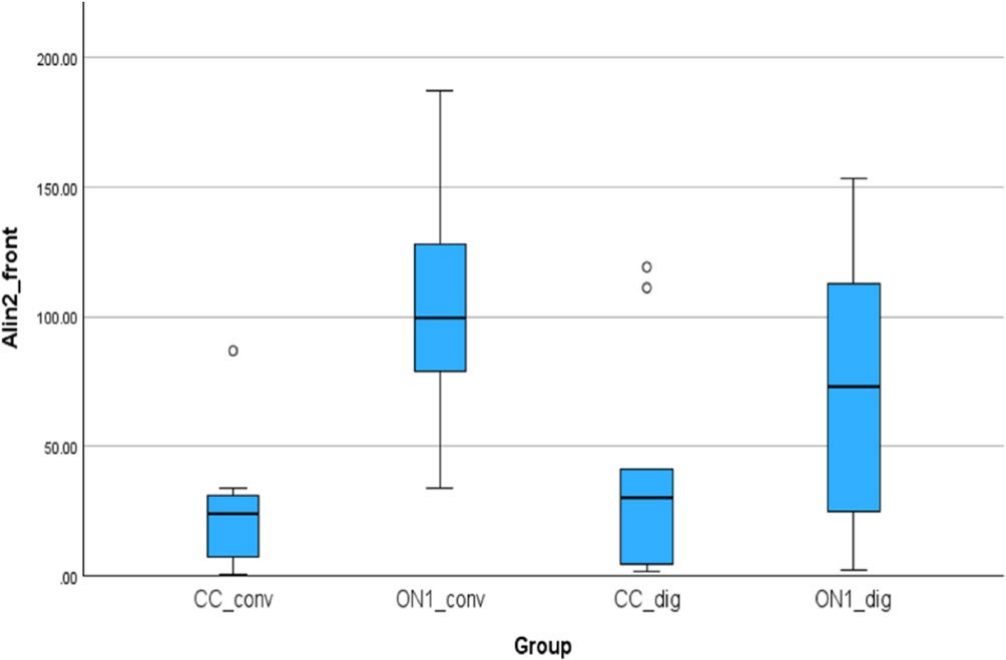
Independent sample Kruskal‐Wallis test of the absolute linear deviations in Alin2_front distance.

Figure 13 shows the absolute linear deviations measured between the analogs in the right and left canine positions. The absolute mean (SD) was 24.90 (25, 19) μm for the CC_conv, 103.86 (41.80) μm for the On1_conv, 40.07 (42.16) μm for the CC_dig, 74.14 (49.34) μm for the On1_dig. Using the nonparametric Absolut‐Kruskal‐Wallis test, a statistically significant difference was observed between the On1_conv and CC_conv groups with a *p*‐value of 0.002 and between the CC_dig and On1_conv groups with a *p*‐value of 0.04.

### 3.5 Accuracy Outcomes—Lin4_diagonal, Alin4_diagonal

**FIGURE 14:**
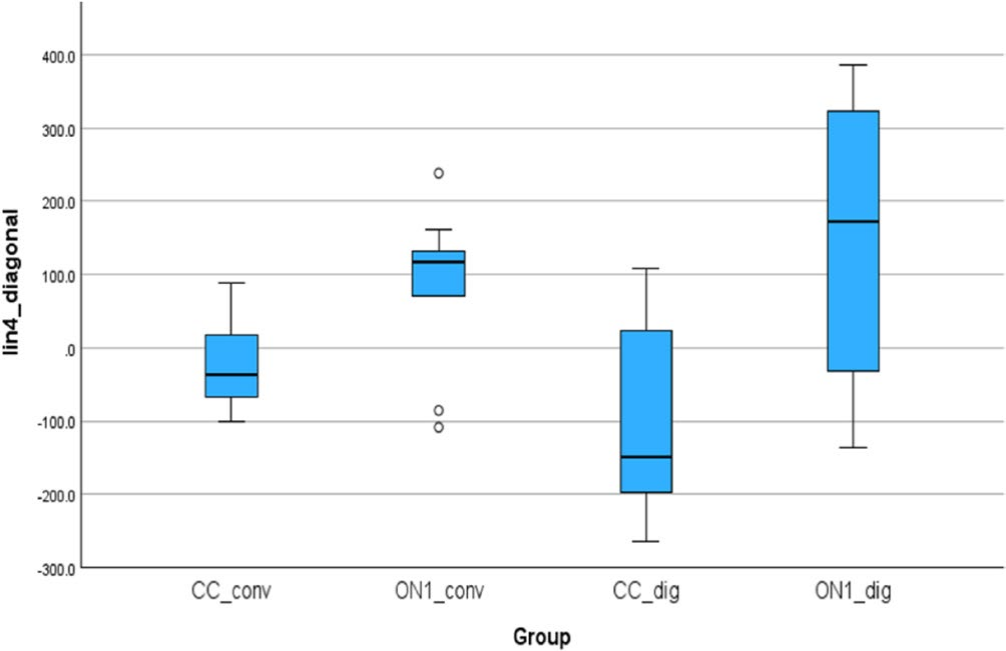
Independent sample Kruskal‐Wallis test of the linear deviations in Lin4_diagonal distance.

Figure 14 shows the linear deviations between the analogs in the right and left molar positions. The mean (SD) was −24.49 (58.20) μm for the CC_conv, 87.46 (106.70) μm for the On1_conv, −104.76 (125.83) for the CC_dig, 140.640 (190.56) μm for the On1_dig.

Using the nonparametric Kruskal‐Wallis test, a statistically significant difference was observed between On1_conv and CC_dig groups with a *p*‐value of 0.02 and between CC_dig and On1_dig with a *p*‐value of 0.01.

**FIGURE 15:**
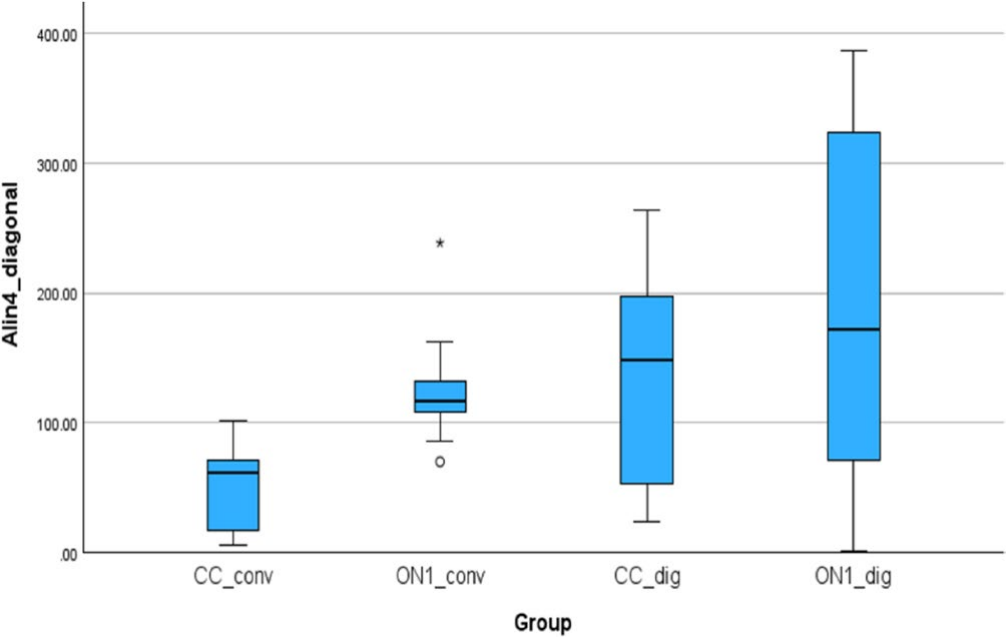
Independent sample Kruskal‐Wallis test of the absolute linear deviations in Alin4_diagonal distance.

Figure 15 shows the absolute linear deviations measured between the analogs in the right and left molar positions. The absolute mean (SD) was 51.29 (33.63) μm for the CC_conv, 126.26 (46.61) μm for the On1_conv, 138.10 (82.68) μm for the CC_dig, 188.86 (136.60) μm for the On1_dig. Using the nonparametric Absolut‐Kruskal‐Wallis test, a statistically significant difference was observed between On1_dig and CC_conv groups with a *p*‐value of 0.009.

## 4 Discussion

This study investigated the accuracy of different implant impression‐making and cast‐fabrication methods using conventional replaceable and coded healing abutment systems. We aimed to provide accuracy data helping clinicians to choose the most accurate workflows for their patients in lower all‐on‐4 cases.

We hypothesized that platform‐level conventional impression‐making combined with gypsum cast‐fabrication is the most accurate method. Our results show statistically significant differences between the digital and conventional methods and also within each workflow, indicating that our original hypothesis is confirmed. However, the clinical relevance of these differences needs to be addressed in more detail.

Mouhyi et al. investigated coded healing abutments in a retrospective clinical study including 103 patients with 126 implant‐supported zirconia restorations, including full‐arch cases as well. Within the limitations of the retrospective study design and short follow‐up time, they found that these systems provided reliable accuracy while promoting hard‐ and soft‐tissue stability [12]. Further studies on a larger sample of patients and a longer follow‐up period are needed to confirm these preliminary clinical outcomes.

In implant prosthodontics, clinicians strive for the highest possible accuracy in all cases.

Although several accuracy ranges have been described [15], there is no consensus to date regarding the clinically acceptable prostheses' accuracy and fit.

The open‐tray impression‐making method is traditionally considered the gold standard in implant prosthodontics [1]. This method usually includes splinting the copings and using high‐precision impression materials such as polyvinyl siloxane or polyether to achieve high accuracy. The conventional impressions are then sent to the dental laboratory and are usually poured with type IV dental stone.

Conventional impression‐making can be done via different methods, such as open‐ or closed‐tray techniques. The impression copings can be attached on implant level, abutment level, or, in the case of coded healing abutment systems, platform level.

The methods mentioned above are complex and time‐consuming processes with several possibilities for errors during and after impression making and cast fabrication.

Digital impression‐making is a popular and promising alternative to these problems. It causes no pain and less discomfort for the patient. According to Gherlone et al., it can be 2–3 times faster than the conventional method [2].

Impression‐making can be an unpleasant process for patients, and according to studies and surveys, they tend to choose digital impression‐making over the conventional method [16].

Digital workflow often eliminates the need for a physical cast, allowing the restoration to be designed directly using the intraoral scan file. Since each clinical step carries the potential for mistakes, reducing the number of clinical steps can increase the overall accuracy of a workflow [8]. However, certain cases still require a physical cast; for example, the precise fitting of contact points, individual dying and finishing of the prostheses, and occlusal corrections [17]. A significant disadvantage of model‐free workflows is the lack of inspection and control on the cast.

Although in some cases, models are necessary, the dentures are not usually fabricated on the cast but on the digital scan files. Still, we decided to include additive cast‐fabrication in the digital workflow to recreate the same clinical and laboratory working steps in both groups.

Five outcomes were measured to investigate the accuracy of different impression‐making methods in lower all‐on‐4 cases: four linear distances between the scanbodies and the RMS deviations of the scanbodies. The linear distance analyses were conducted using both signed and absolute deviations. In the case of large positive and negative deviations, the resulting mean values might be close to zero, suggesting a false slight overall deviation. However, the direction of the deviations has no clinical relevance; only the fit matters. Conducting absolute deviation analyses can eliminate these false results and represent the true extent of the deviations.

Root mean square deviation is a statistical measure used to quantify the accuracy of virtual models compared to a reference. In dental research, RMS deviation is often used to assess the trueness and precision of different impression‐making methods, prosthetic designs, or treatment simulations by calculating the average magnitude of point‐wise discrepancies across surfaces. Lower RMS values indicate higher accuracy and better model agreement [18, 19].

Using the best‐fit alignment method and analyzing RMS deviations has considerable clinical relevance in cases of full‐arch prostheses because it represents the same errors that can influence the passive fit of large, one‐piece prostheses [20].

For alignment, we selected the mucosal surface and excluded the surface of the scanbodies. If the scanbodies are included in the alignment process, the deviations between them might change since best‐fit will try to bring the surfaces closer to each other. This process might mask the actual deviations, creating a more uniformly distributed and overall smaller deviation.

Based on the RMS deviations, the conventional, open‐tray impression‐making method and gypsum cast‐fabrication were significantly more accurate than the intraoral scanning and additive cast‐fabrication. The level of significance was outstanding (*p* = 0.00005). According to these results, the conventional workflow is far superior to the digital one. However, the linear distance deviations show a less clear picture.

In clinical practice, impression‐making and fabrication of all‐on‐4 prostheses are usually performed on the abutment level, not the implant level [21]. However, our master cast serves the purpose of including several clinical situations at the same time as well, such as a lateral bridge supported by two implants, implants with significant angular discrepancies, implants parallel to each other, and multiple implants placed in the completely edentulous jaw.

The four linear distances each have their own importance for conventional impression‐making and intraoral scanning.

Lin1_right is the distance between the right lower second molar and the right lower canine implant position. This is the site where the digital impression‐making was started, and the two implants are at an angle of approximately 30°.

Lin2_front is the distance between the right lower canine and the left lower canine implant positions. At this site, the two implants are parallel to each other.

Lin3_left is the distance between the left lower second molar and the left lower canine implant position. The scanning was finished at this site, which means the cumulative errors are supposedly higher than on the right side [22]. The position and angulation of the two implants are the same as on the right side. This should mean no difference between the two sides in the conventional method.

Lin4_diagonal is the distance between the two molar positions across the molar zones. This distance represents the overall distortion and warping of the models.

Lin1_right and Lin3_left have approximately the same baseline distance, the same angulation, and the same position. For the conventional methods, there should be little to no difference between the two sides. However, our results do not follow this hypothesis since Lin1_right CC_Conv and ON1_conv have values of 117 and −7, and Lin3_left has the same values measured at 24 and 53. The low number of repetitions might explain this difference between the two sites.

In the case of angulated implants (above 15°), the open‐tray impression technique and the splinting of the copings are the recommended conventional techniques [23]. The impression materials used for implant impressions should have sufficient rigidity to keep the copings stable during and after the impression‐making. The most preferred materials are polyether and vinyl poly‐siloxane [23, 24]. In our study, vinyl poly‐siloxane material was used.

Splinting of the copings helps increase the positional accuracy and stability of the impression copings. This is especially important when removing the completely set impression and fastening the implant analogs to the copings since these two phases create the most strain and torque [25, 26]. Recent studies show that the splinted impression technique provides significantly better accuracy in the case of completely edentulous jaws with 4 to 6 implants [27].

The digital impression technique utilizes optical principles to image the position of the implant scanbodies, thus determining the position of the implants. Unlike the conventional impression, this has no physical effects on the scanbodies. This means that unlike in the conventional method, the inaccuracies of the digital workflow do not come from physical effects such as tensions, strain, or shrinkage. Accordingly, the implant position and angulation do not make scanning more challenging in the way that they make impression‐making.

However, for an accurate digital impression, the whole surface of the scanbody should be completely visible, which might not be possible in the case of large angulations. Incomplete surfaces of the scanbodies might hinder the matching process during the laboratory design phase [28].

In the digital method, however, a more significant difference is expected between the two ends of the jaw. According to literature data, inaccuracies add up to the far end of the jaw during scanning [22, 29]. Our results support these observations with −137 and 97 for lin1_right and −147 and 141 for lin3_left.

Lin2_front is a different case since these implants are parallel to each other. This supposedly has more relevance for the conventional method since the rigid impression materials act differently in the case of angulated and parallel implants [30].

The shape and size of the scanbodies also play a vital role in scanning accuracy, especially in the alignment during digital design processes. The shorter On1 healing C cap is much harder to recognize and precisely align than the longer Elos scanbody with a distinct angulated edge. The operator responsible for the digital model design reported difficulties in the case of On1 alignment. This follows our results: In 3 out of 4 cases, the On1_dig group had worse accuracy values than the CC_dig group.

Our study has several limitations that must be addressed. Accuracy was assessed under in vitro conditions, without considering clinical factors such as saliva, blood, or soft tissue movement. While the sample size was sufficient for this study, a larger sample could provide more universally applicable results. Additionally, we measured the combined accuracy of impression‐making and cast fabrication to simulate a clinical scenario, meaning our results reflect the total deviations from both processes. As a result, it is not possible to distinguish the individual contributions of each procedure separately. The accuracy of additive cast fabrication varies significantly depending on factors such as material choice, printing settings, and post‐processing [31]. These variables can only be thoroughly analyzed in studies that focus on each parameter separately.

## 5 Conclusion

The following conclusions were drawn on data measured under in vitro conditions on a lower jaw model containing four implants in an all‐on‐4 position:

1. Based on the RMS deviations of the implant analogs, the conventional method is significantly more accurate at both the implant‐and platform levels.
2. The RMS deviations of the implant analogs are smaller at the platform level with both conventional and digital methods.
3. During intraoral scanning, inaccuracies add up to larger deviations at the far end of the jaw.
4. Digital method is more reliable in angulated implants, but the conventional method is more accurate in the case of parallel implants.

## 6 Implication for Research

The scientific value of accuracy studies could be greatly increased by more in vivo research. The clinical relevance of accuracy data should be studied, and acceptability thresholds should be determined. Further studies are needed to assess the influencing factors of both conventional and digital workflow.

## 7 Implications for Practice

Our findings are based on in vitro data, which have limited clinical value because of the lack of clinical factors (such as the presence of saliva or blood, ambient light conditions, anatomical variances, and patient movements). Although accuracy is one of the most crucial factors, the advantages and disadvantages of either method should be considered thoroughly. Based on our data, intraoral scanning is not recommended for full‐arch prostheses.

## Author Contributions

**Boldizsár Vánkos:** conceptualization, investigation, writing – original draft. **Dénes Palaszkó:** resources, investigation. **Kata Kelemen:** methodology, validation. **Anna Németh:** conceptualization. **Judit Schmalzl:** methodology, investigation. **Dániel Márk Zentai:** resources, investigation. **Elek Dinya:** conceptualization, formal analysis, data curation. **Kata Kelemen:** conceptualization, methodology, validation, supervision, writing – review and editing. **Péter Hermann:** project administration, supervision. **Barbara Kispélyi:** project administration, supervision, writing – review and editing. All authors certify that they have participated sufficiently in the work to take public responsibility for the content, including participation in the manuscript's concept, design, analysis, writing, or revision.

## Conflicts of Interest

The authors declare no conflicts of interest.

## Data Availability

Raw data is available on request.
